# Sleep Duration and Daytime Napping and Risk of Type 2 Diabetes Among Japanese Men and Women: The Japan Collaborative Cohort Study for Evaluation of Cancer Risk

**DOI:** 10.2188/jea.JE20220118

**Published:** 2023-11-05

**Authors:** Reiko Okada, Masayuki Teramoto, Isao Muraki, Akiko Tamakoshi, Hiroyasu Iso

**Affiliations:** 1 Department of Social Medicine, Osaka University Graduate School of Medicine, Osaka, Japan; 2 Department of Public Health, Graduate School of Medicine, Hokkaido University, Sapporo, Japan; 3 Institute for Global Health Policy Research, Bureau of International Health Cooperation, National Center for Global Health and Medicine, Tokyo, Japan

**Keywords:** sleep duration, napping, type 2 diabetes, cohort study

## Abstract

**Background:**

Little is known about the impacts of sleep duration and daytime napping on the risk of type 2 diabetes mellitus (T2DM).

**Methods:**

In this study, 20,318 participants (7,597 men, 12,721 women) aged 40–79 years without a history of T2DM, stroke, coronary heart disease, or cancer at baseline (1988–1990), completed the baseline survey and the 5-year follow-up questionnaires, which included average sleep duration, napping habits, and self-reports of physician-diagnosed diabetes. The multivariable odds ratios (ORs) with 95% confidence intervals (CIs) were calculated using a logistic regression model.

**Results:**

During the 5-year follow-up, 531 new cases of T2DM (266 men and 265 women) were documented. Sleep duration ≥10 hours was associated with higher risk of T2DM compared to sleep duration of 7 hours (OR 1.99; 95% CI, 1.28–3.08). The excess risk was observed for both sexes and primarily found among the non-overweight; the multivariable ORs of sleeping ≥10 hours compared to 7 hours were 2.05 (95% CI, 1.26–3.35) for the non-overweight (BMI <25 kg/m^2^) and 1.38 (95% CI, 0.49–3.83) for the overweight (BMI ≥25 kg/m^2^). The respective ORs of nappers versus non-nappers were 1.30 (95% CI, 1.03–1.63) and 0.92 (95% CI, 0.65–1.29). Among the non-overweight, nappers who slept ≥10 hours had the highest risk of T2DM (OR 2.84; 95% CI, 1.57–5.14), non-nappers who slept ≥10 hours (OR 2.27; 95% CI, 1.27–4.06), and nappers who slept <10 hours (OR 1.30; 95% CI, 1.03–1.64), compared with non-nappers who slept <10 hours.

**Conclusion:**

Long sleep duration was associated with the risk of T2DM in both sexes, which was confined to the non-overweight.

## INTRODUCTION

Type 2 diabetes (T2DM) is one of the top ten causes of death in adults and was attributable to four million deaths in 2017.^1^ The global incidence of T2DM is increasing every year and is projected to reach 10.9% by 2045, up from 9.3% in 2019.^1^ Diabetes can damage several organs and cause various diseases, such as diabetic neuropathy, diabetic retinopathy, diabetic nephrotic, and cardiovascular disorders, resulting in high mortality and morbidity.^2^ According to the American Diabetes Association, the cost of treatment for T2DM reached $327.2 billion in 2017^3^; therefore, prevention of T2DM is urgent.

Sleep duration has been associated with risks of mortality from all causes,^4^^,^^5^ cardiovascular diseases,^6^^,^^7^ or stroke.^8^ Several meta-analyses on the risks of T2DM have also reported a U-shaped association with 7–8 hours of sleep associated with the lowest risk of T2DM.^9^^–^^11^

According to a recent meta-analysis, habitual nappers were at a higher risk of all-cause mortality, while no significant association was found between daytime napping and cardiovascular disease.^12^ Daytime napping for more than 1 hour was also associated with an increased risk of T2DM compared to non-nappers, in a meta-analysis of four cross-sectional and six cohort studies.^13^ However, in that meta-analysis, significant heterogeneity of the association between daytime napping and the risk of T2DM was observed: a positive association in some studies^14^^–^^16^ and no association in others.^17^^–^^19^

In a Finnish prospective study that involved 12,244 twin men and women aged 30–66 years with a 14-year follow-up, habitual nappers were at an increased risk of T2DM compared to non-nappers. However, after additionally adjusting for body mass index (BMI), the association became insignificant, suggesting that obesity could explain the association between daytime napping and diabetes incidence.^18^ Other studies showed positive association after further adjustment for BMI.^14^^–^^16^^,^^20^

Furthermore, a previous study has reported an interaction between sleep duration and daytime napping on the risk of T2DM.^14^ In particular, since Japanese people have a particularly short average sleep time compared to other countries,^21^ daytime napping may compensate for lack of sleep duration.

Therefore, we aimed to examine whether sleep duration and daytime napping are associated with the risk of T2DM, independent of BMI, and the combined impact of sleep duration and daytime napping on the risk of T2DM in Japanese men and women.

## METHODS

The data that support the findings of this study are available upon reasonable request.

### Study population and baseline questionnaires

The Japan Collaborative Cohort Study for Evaluation of Cancer Risk (JACC) is a nationwide community-based prospective study that started in 1988–1990. A total of 110,585 participants (46,395 men and 64,190 women) aged 40–79 years living in 45 communities across Japan participated in the study. The methodology of the study has been described elsewhere.^22^ Briefly, self-administered questionnaires were used to collect baseline information including demographic characteristics, medical history, and lifestyle.

Informed consent was provided by the participants or community representatives in order to participate in this study. Individual informed consent was obtained from each participant in 36 of the 45 study areas, and group consent was obtained from the leader of each area in the remaining nine areas. The JACC protocol was approved by the Hokkaido University, Nagoya University, and Osaka University ethics committees.

### Follow-up survey

We conducted a 5-year follow-up survey in 31 of 45 communities, and 46,540 participants completed the 5-year follow-up survey. A total of 11,205 participants living in six communities were excluded because questions on sleep duration and daytime napping habits were not included in the questionnaire. We also excluded 3,052 participants who did not report baseline information on sleep duration and daytime napping habits. We excluded 7,706 participants whose data on T2DM both at baseline and 5-year follow-up surveys were unavailable. Finally, we excluded 2,169 night or shift workers and 2,090 participants with a history of diabetes, stroke, coronary heart disease, or cancer at baseline. A total of 20,318 participants (7,597 men and 12,721 women) were included in the analyses.

### Sleep duration and daytime napping

The average sleep duration on weekdays during the preceding years was obtained through questions on general health status. The average sleep duration per day was classified into six categories based on self-reported responses: ≤5, 6, 7, 8, 9, and ≥10 hours. Fractions were rounded off (eg, 7 hours represented responses from 6.5 to 7.4 hours). Regarding daytime napping habits, we asked the participants, “Do you take a daytime nap?” with the following possible answers: “yes” or “no.”

### Assessment of covariates

Self-administered baseline questionnaire was used to collect the information on demographic and lifestyle covariates, such as age, sex, height, weight, history of hypertension, family history of diabetes, smoking status, alcohol consumption, exercise and walking habits, educational level, perceived mental status, occupation, and eating habits. BMI was calculated as body weight (kg) divided by height squared (m^2^).

### Ascertainment of diabetes

We defined self-reported physician-diagnosed diabetes at the 5-year follow-up survey as T2DM cases. The validity of self-reported T2DM cases was confirmed by comparing self-reported diabetes with the laboratory findings or treatment status among 1,230 men and 1,837 women in one of the JACC study communities.^23^ The definition of T2DM was ≥7.8 mmol/L (≥140 mg/dL) of fasting serum glucose concentration or ≥11.1 mmol/L (≥200 mg/dL) of casual serum glucose concentration, or treatment with oral hypoglycemic agents or insulin. The sensitivity and specificity of self-reporting were 70% and 95% for men and 75% and 98% for women, respectively.

### Statistical analysis

Age-adjusted mean values and prevalence of risk factors for T2DM calculated for the categories of sleep duration, daytime napping, and their combination. The odds ratios (ORs) with 95% confidence intervals (CIs) of the risk of T2DM according to sleep duration and daytime napping were estimated using a logistic regression model after adjustment for potential confounding factors. We then examined the combined effects of sleep duration and daytime napping by classifying participants into four groups: non-nappers with sleep duration less than 10 hours (reference), nappers with sleep duration less than 10 hours, non-nappers with sleep duration of 10 hours or more, and nappers with sleep duration of 10 hours or more. We then repeated the analyses, stratifying by BMI (<25 or ≥25 kg/m^2^) and by age (<65 or ≥65 years) to examine whether the association can be explained by confounding due to BMI and age. The potential confounding factors were age (continuous), sex (women or men), BMI (sex-specific quintile), a history of hypertension (yes or no), family history of diabetes (yes or no), smoking status (never, ex-smoker, or current smoker of 1–19 or current smoker of ≥20 cigarettes per day), alcohol consumption (never drinker, ex-drinker, or current drinker of 0.1–45.9 g or ≥46.0 g ethanol per day), hours of exercise (almost never, 1–4 h, or ≥5 h per week), hours of walking (almost never, 0.5 h, or >0.5 h per day), perceived mental stress (low, moderate, or high), educational level (≤18 years or ≥19 years of age on completion of education), employment status (unemployed or employed), green tea intake (rarely, 3–4 cups/week, 1–4 cups/day, and ≥5 cups/day), coffee intake (rarely, 1–6 cups/week, 1–2 cups/day, and >2 cups/day) and frequency of vegetable, fruit, soybean and fish intake (quintile). The SAS version 9.4 (SAS, Inc., Cary, NC, USA) was used for all statistical analyses. All statistical tests were two-sided, and *P* values <0.05 were considered statistically significant.

## RESULTS

Table 1 shows the age-adjusted mean values and prevalence of risk characteristics at baseline according to the categories of sleep duration, daytime napping, and their combination. Participants with a sleep duration of ≥10 hours were more likely to be older, current smokers, current drinkers, unemployed, daytime nappers, and were less likely to be men and to have history of hypertension, and perceived high mental stress, higher education, exercise, and intakes of green tea, coffee and fruits, compared with those with a sleep duration of 7 hours. Similar trends were observed for nappers compared with non-nappers, and for nappers with a sleep duration of ≥10 hours compared with non-nappers with a sleep duration of <10 hours.

**Table 1.** Age-adjusted baseline characteristics of participants according to sleep duration and daytime napping

	Sleep duration (hours/day)						Daytime napping		Combination of daytime napping and sleep duration			
		
	≤5	6	7	8	9	≥10	Non- nappers	Nappers	Non-nappers + <10 hours of sleep	Nappers + <10 hours of sleep	Non-nappers + ≥10 hours of sleep	Nappers + ≥10 hours of sleep
Number of participants	803	3,553	7,809	6,605	1,128	420	13,876	6,442	13,689	6,215	187	227
Age, years	58.4	55.2	54.6	57.1	60.1	65.4	55.4	57.9	55.3	57.6	64.7	66.0
Sex, % of men	75.8	74.3	67.1	53.4	45.0	45.2	65.9	55.6	66.2	56.0	45.3	45.0
Body mass index, kg/m^2^	22.7	22.7	22.6	22.8	22.7	22.4	22.6	22.9	22.6	22.9	22.3	22.6
History of hypertension, %	17.2	16.1	16.3	16.5	15.9	14.5	15.2	18.6	15.3	18.6	11.8	17.6
Family history of diabetes, %	11.0	11.0	10.6	10.2	9.3	10.6	10.7	10.2	10.7	10.1	8.9	11.4
Current smoker, %	13.5	15.5	19.8	27.1	33.8	35.3	21.4	24.1	21.2	23.8	38.6	32.8
Current drinker, %	34.9	35.3	38.8	47.3	51.4	49.1	39.2	47.2	39.0	47.1	48.9	49.4
High mental stress, %	32.3	24.2	20.3	17.0	18.4	19.8	22.0	16.6	22.0	16.4	18.5	20.9
College or higher education, %	12.4	14.0	13.9	13.5	10.3	7.8	14.5	11.2	14.6	11.3	6.5	8.3
Unemployed, %	21.2	16.0	16.0	17.2	22.2	30.5	16.8	18.2	16.6	17.7	23.4	35.8
Walking ≥1 h/day, %	50.5	53.4	53.8	54.8	59.4	55.4	52.2	58.7	52.1	58.9	59.2	52.5
Exercise ≥1 h/week, %	22.8	26.2	26.1	25.8	26.2	23.6	25.0	27.7	25.1	27.7	19.9	27.8
Green tea intake ≥1 cup/day, %	74.5	76.8	76.6	76.8	75.9	73.3	77.8	73.8	77.7	74.0	80.0	68.2
Coffee intake ≥1 cup/day, %	45.2	46.1	43.8	37.9	32.1	35.6	45.4	33.3	45.5	33.3	37.9	33.8
Vegetable intake, times/week	14.2	14.4	14.4	14.5	13.7	14.6	14.0	15.2	14.0	15.2	14.0	15.3
Fish intake, times/week	6.2	6.5	6.7	7.0	7.1	6.7	6.5	7.2	6.5	7.2	6.5	6.9
Fruits intake, times/week	7.1	7.5	7.5	7.0	6.3	6.5	7.4	6.8	7.4	6.8	6.7	6.3
Soybeans intake, times/week	4.7	4.8	5.0	5.0	4.6	4.8	4.9	5.1	4.9	5.1	4.6	5.1
Napping, %	22.3	25.8	28.5	36.5	44.0	50.2	—	—				
Sleep duration, hours	—	—	—	—	—	—	7.1	7.4				

Data are mean for continuous variables and percentages for categorical variables.

During the 5-year follow-up survey, we observed 531 new cases of T2DM (266 men and 265 women). Table 2 shows that a sleep duration of ≥10 hours was associated with a higher risk of T2DM compared to a sleep duration of 7 hours in the age- and sex-adjusted analyses. These associations remained statistically significant after further adjustment for potential confounding factors; the multivariable ORs of T2DM compared with sleep duration of 7 hours were 1.11 (95% CI, 0.71–1.74) for ≤5 hours, 0.92 (95% CI, 0.71–1.20) for 6 hours, 0.87 (95% CI, 0.70–1.07) for 8 hours, 1.05 (95% CI, 0.73–1.53) for 9 hours, and 1.98 (95% CI, 1.28–3.08) for ≥10 hours. Similar associations were observed between men and women; the multivariable ORs for sleep duration of ≥10 hours compared to that of 7 hours were 1.85 (95% CI, 1.03–3.34) in men and 2.60 (95% CI, 1.33–5.09) in women. Shorter sleep duration of 6 hours and ≤5 hours were not associated with the risk of T2DM, although the non-significant excess risk was observed for men.

**Table 2.** Odds ratios and 95% confidence intervals of risk of type 2 diabetes according to sleep duration

	Sleep duration (hours/day)					

	≤5	6	7	8	9	≥10
Total						
Number of participants	803	3,553	7,809	6,605	1,128	420
Number of cases	23	83	193	167	37	28
Age- and sex-adjusted OR (95% CI)	1.11 (0.71–1.73)	0.96 (0.74–1.25)	Ref	0.90 (0.73–1.11)	1.05 (0.73–1.51)	1.98 (1.30–3.02)
Multivariable OR (95% CI)	1.11 (0.71–1.74)	0.92 (0.71–1.20)	Ref	0.87 (0.70–1.07)	1.05 (0.73–1.53)	1.99 (1.28–3.08)
Men						
Number of participants	194	913	2,565	3,075	620	230
Number of cases	11	40	84	91	24	16
Age- adjusted OR (95% CI)	1.61 (0.84–3.07)	1.31 (0.89–1.92)	Ref	0.87 (0.64–1.17)	1.08 (0.68–1.72)	1.83 (1.04–3.21)
Multivariable OR (95% CI)	1.63 (0.84–3.19)	1.26 (0.85–1.86)	Ref	0.85 (0.63–1.16)	1.08 (0.67–1.74)	1.85 (1.03–3.34)
Women						
Number of participants	609	2,640	5,244	3,530	508	190
Number of cases	12	43	109	76	13	12
Age-adjusted OR (95% CI)	0.86 (0.47–1.58)	0.77 (0.54–1.11)	Ref	0.96 (0.71–1.30)	1.06 (0.59–1.91)	2.34 (1.24–4.43)
Multivariable OR (95% CI)	0.86 (0.47–1.59)	0.73 (0.51–1.05)	Ref	0.90 (0.66–1.22)	1.09 (0.60–2.00)	2.60 (1.33–5.09)

CI, confidence interval; OR, odds ratio.

Multivariable OR: adjusted for age, sex, daytime napping, history of hypertension, body mass index, smoking status, alcohol consumption, hours of exercise, hours of walking, educational level, perceived mental stress, regular employment, coffee intake, green tea intake, dietary intakes of vegetable, fish, fruits and soybeans and family history of diabetes.

As shown in Table 3, daytime napping tended to be positively associated with an increased risk of T2DM, but the associations did not reach statistical significance in both sexes.

**Table 3.** Odds ratios and 95% confidence intervals of risk of type 2 diabetes according to daytime napping

	Non-nappers	Nappers
Total		
Number of participants	13,876	6,442
Number of cases	331	200
Age- and sex-adjusted OR (95% CI)	Ref	1.17 (0.97–1.40)
Multivariable OR (95% CI)	Ref	1.14 (0.95–1.38)
Men		
Number of participants	4,728	2,869
Number of cases	159	107
Age-adjusted OR (95% CI)	Ref	1.05 (0.82–1.35)
Multivariable OR (95% CI)	Ref	1.10 (0.84–1.43)
Women		
Number of participants	9,148	3,573
Number of cases	172	93
Age-adjusted OR (95% CI)	Ref	1.31 (1.01–1.69)
Multivariable OR (95% CI)	Ref	1.20 (0.92–1.58)

CI, confidence interval; OR, odds ratio.

Multivariable OR: adjusted for age, sex, sleep duration, history of hypertension, body mass index, smoking status, alcohol consumption, hours of exercise, hours of walking, educational level, perceived mental stress, regular employment, coffee intake, green tea intake and dietary intakes of vegetable, fish, fruits and soybeans and family history of diabetes.

In the age-stratified analyses, sleep duration tended to be positively associated with the risk of T2DM in persons aged <65 years, while the association was J-shaped in persons aged ≥65 years, where the excess risk of borderline statistical significance was observed for shorter sleep duration (Table 4). The excess risk of T2DM for long sleep duration and daytime napping was primarily found among the non-overweight; the multivariable ORs for sleep duration of ≥10 hours were 2.05 (95% CI, 1.26–3.35) in the non-overweight and 1.38 (95% CI, 0.49–3.83) in the overweight, compared to sleeping duration of 7 hours. The corresponding ORs for daytime napping were 1.30 (95% CI, 1.03–1.63) and 0.92 (95% CI, 0.65–1.29) compared to non-nappers.

**Table 4.** Odds ratios and 95% confidence intervals of risk of type 2 diabetes according to daytime napping stratified by age and body mass index

	Sleep duration (hours/day)						Non-nappers	Nappers

	≤5	6	7	8	9	≥10		
Age, years								
<65								
Number of participants	583	2,955	6,692	5,176	753	180	11,539	4,800
Number of cases	14	61	170	125	22	9	258	143
Multivariable OR (95% CI)	0.94 (0.54–1.64)	0.79 (0.59–1.07)	Ref	0.77 (0.61–0.99)	0.88 (0.56–1.41)	1.51 (0.74–3.10)	Ref	1.22 (0.98–1.52)
≥65								
Number of participants	220	598	1,117	1,429	375	240	2,237	1,642
Number of cases	9	22	23	42	15	19	73	57
Multivariable OR (95% CI)	2.08 (0.93–4.68)	1.85 (1.01–3.39)	Ref	1.45 (0.86–2.45)	2.11 (1.06–4.21)	4.38 (2.23–8.60)	Ref	0.96 (0.66–1.40)
BMI, kg/m^2^								
<25.0								
Number of participants	653	2,876	6,452	5,363	928	361	11,430	5,203
Number of cases	14	47	126	111	27	23	206	142
Multivariable OR (95% CI)	0.99 (0.56–1.75)	0.86 (0.61–1.20)	Ref	0.87 (0.67–1.14)	1.10 (0.71–1.70)	2.05 (1.26–3.35)	Ref	1.30 (1.03–1.63)
≥25.0								
Number of participants	150	677	1,357	1,242	200	59	2,446	1,239
Number of cases	9	36	67	56	10	5	125	58
Multivariable OR (95% CI)	1.24 (0.59–2.56)	1.04 (0.68–1.59)	Ref	0.84 (0.57–1.22)	1.00 (0.49–2.04)	1.38 (0.49–3.83)	Ref	0.92 (0.65–1.29)

BMI, body mass index; CI, confidence interval; OR, odds ratio.

Multivariable OR: adjusted for age, sex, history of hypertension, smoking status, alcohol consumption, hours of exercise, hours of walking, educational level, perceived mental stress, regular employment, coffee intake, green tea intake and dietary intakes of vegetable, fish, fruits and soybeans, family history of diabetes and mutually for sleep duration and daytime napping.

As shown in Table 5, compared to non-nappers with a sleep duration of <10 hours, nappers with a sleep duration of ≥10 hours were at a higher risk of T2DM. The multivariable ORs for nappers with the sleep duration of ≥10 hours were 2.62 (95% CI, 1.55–4.43) in total participants, 2.51 (95% CI, 1.27–5.00) in men, and 3.23 (95% CI, 1.39–7.50) in women. The excess risk for non-nappers with sleep duration of ≥10 hours was observed in women: the multivariable OR was 3.26 (95% CI, 1.25–8.49).

**Table 5.** Odds ratios and 95% confidence intervals of risk of type 2 diabetes according to the combination of daytime napping and sleep duration

	Non-nappers + <10 hours of sleep	Nappers + <10 hours of sleep	Non-nappers + ≥10 hours of sleep	Nappers + ≥10 hours of sleep
Total				
Number of participants	13,689	6,215	187	227
Number of cases	321	182	10	18
Age- and sex-adjusted OR (95% CI)	Ref	1.13 (0.94–1.36)	1.74 (0.90–3.34)	2.56 (1.55–4.25)
Multivariable OR (95% CI)	Ref	1.13 (0.93–1.36)	1.87 (0.96–3.65)	2.62 (1.55–4.43)
Men				
Number of participants	4,625	2,743	103	126
Number of cases	154	96	5	11
Age-adjusted OR (95% CI)	Ref	1.00 (0.77–1.30)	1.27 (0.51–3.17)	2.28 (1.19–4.36)
Multivariable OR (95% CI)	Ref	1.04 (0.79–1.37)	1.27 (0.49–3.26)	2.51 (1.27–5.00)
Women				
Number of participants	9,064	3,472	84	101
Number of cases	167	86	5	7
Age-adjusted OR (95% CI)	Ref	1.28 (0.98–1.67)	2.59 (1.02–6.55)	3.01 (1.35–6.70)
Multivariable OR (95% CI)	Ref	1.22 (0.93–1.61)	3.26 (1.25–8.49)	3.23 (1.39–7.50)

CI, confidence interval; OR, odds ratio.

Multivariable OR: adjusted for age, sex, history of hypertension, body mass index, smoking status, alcohol consumption, hours of exercise, hours of walking, educational level, perceived mental stress, regular employment, coffee intake, green tea intake and dietary intakes of vegetable, fish, fruits and soybeans and family history of diabetes.

The excess risk of T2DM for nappers with a sleep duration of ≥10 hours was observed in persons aged ≥65 and in the non-overweight (Table 6). The multivariable ORs for nappers with a sleep duration of ≥10 hours compared to non-nappers with a sleep duration of <10 hours were 3.14 (95% CI, 1.60–6.18) in older ages, and 2.84 (95% CI, 1.57–5.14) in the non-overweight. The excess risk for non-nappers with sleep duration of ≥10 hours was in the non-overweight: the multivariable OR was 2.27 (95% CI, 1.12–4.06).

**Table 6.** Odds ratios and 95% confidence intervals of risk of type 2 diabetes according to the combination of daytime napping and sleep duration stratified by age and body mass index

	Non-nappers + <10 hours of sleep	Nappers + <10 hours of sleep	Non-nappers + ≥10 hours of sleep	Nappers + ≥10 hours of sleep
Age, years				
<65				
Number of participants	11,452	4,710	87	90
Number of cases	254	138	4	5
Age-adjusted OR (95% CI)	Ref	1.21 (0.97–1.49)	1.57 (0.57–4.34)	1.92 (0.77–4.81)
Multivariable OR (95% CI)	Ref	1.21 (0.97–1.50)	1.74 (0.61–4.92)	2.19 (0.85–5.62)
≥65				
Number of participants	2,237	1,505	100	137
Number of cases	67	44	6	13
Age-adjusted OR (95% CI)	Ref	0.94 (0.64–1.38)	2.02 (0.85–4.82)	3.20 (1.70–6.02)
Multivariable OR (95% CI)	Ref	0.91 (0.61–1.35)	2.31 (0.94–5.71)	3.14 (1.60–6.18)
BMI, kg/m^2^				
<25.0				
Number of participants	11,266	5,012	164	191
Number of cases	197	128	9	14
Age-adjusted OR (95% CI)	Ref	1.27 (1.01–1.60)	2.18 (1.09–4.38)	2.79 (1.57–4.97)
Multivariable OR (95% CI)	Ref	1.30 (1.03–1.64)	2.27 (1.12–4.60)	2.84 (1.57–5.14)
≥25.0				
Number of participants	2,423	1,203	23	36
Number of cases	124	54	1	4
Age-adjusted OR (95% CI)	Ref	0.83 (0.59–1.15)	0.71 (0.09–5.35)	2.00 (0.69–5.82)
Multivariable OR (95% CI)	Ref	0.88 (0.63–1.25)	0.67 (0.09–5.31)	1.86 (0.59–5.81)

BMI, body mass index; CI, confidence interval; OR, odds ratio.

Multivariable OR: adjusted for age, sex, history of hypertension, body mass index, smoking status, alcohol consumption, hours of exercise, hours of walking, educational level, perceived mental stress, regular employment, coffee intake, green tea intake and dietary intakes of vegetable, fish, fruits and soybeans and family history of diabetes.

## DISCUSSION

In this large prospective study of Japanese men and women aged 40 to 79 years, we found that sleep duration of ≥10 hours was associated with an approximately two-fold increased risk of T2DM compared to 7 hours of sleep in both men and women, and this association was confined to the non-overweight. Shorter sleep duration and daytime napping were not associated with the risk of T2DM, although the excess risk for shorter sleep duration was suggested in men and older ages. Regarding the combined effect of sleep duration and daytime napping, the risks of T2DM were approximately 2.5 to 3 times higher in nappers with the long sleep duration than in non-nappers without it.

Our results are in line with those of previous studies. In a cohort study of 151,691 white, African American, Japanese American, Native Hawaiian, and Latino participants aged 45–75 years with a median 7.9 years of follow-up, long sleep duration (≥9 hours) was associated with a higher risk of diabetes, but not with short sleep duration (≤6 hours) compared to sleep duration of 7–8 hours; the multivariable HRs were 1.12 (95% CI, 1.04–1.21) for long sleepers and 1.04 (95% CI, 0.99–1.09) for short sleepers.^24^ Another cohort study of 27,009 retired Chinese employees with an average age of 62.5 years examined the independent and combined impacts of sleep duration and daytime napping on the risk of T2DM.^17^ In that study, participants who slept 10 hours and more were at a higher risk of T2DM compared with those who slept 7–8 hours; the multivariable HR was 1.42 (95% CI, 1.08–1.87). Similarly, napping of >90 minutes was associated with a higher risk of T2DM compared to non-nappers; the multivariable HR was 1.28 (95% CI, 1.03–1.59). Further, they examined the combined effects of sleep duration and daytime napping (>60 min). The multivariable HR of T2DM was 1.72 (95% CI, 1.03–2.85) for nappers who slept 10 hours or more, compared to non-nappers who slept 7–8 hours.

The excess risk of T2DM for long sleep duration and daytime napping was found in the non-overweight group, while such associations were not observed in the overweight group. A cohort study of 53,916 Chinese participants aged 30–79 years with average BMI of 22.9 (standard deviation, 3.1) kg/m^2^ (24.5% overweight participants) reported that daytime napping was positively associated with the risk of T2DM regardless of BMI; the multivariable HRs adjusting for socio-demographic status, behavioral lifestyles, sleep duration, BMI, waist circumference, and snoring compared to those without daytime napping was 1.39 (95% CI, 1.21–1.59).^19^ In that study, stratification by BMI (<25 kg/m^2^ or ≥25 kg/m^2^) did not change the association materially; the HRs were 1.44 (95% CI, 1.18–1.75) for the non-overweight and 1.35 (95% CI, 1.11–1.63) for the overweight. One of the reasons for the lack of association of prolonged sleep and daytime napping with the risk of T2DM among overweight patients in the present study might be attributable to the smaller number of incident T2DM cases and also its increased absolute risk in the overweight group without prolonged sleep or daytime napping because they had other metabolic risk factors associated with overweight.

The underlying mechanisms of the association between long sleep duration and daytime napping with an increased risk of T2DM among non-overweight individuals are not well known, but several potential mechanisms have been addressed. Compared to normal sleep duration, long sleep duration was associated with increased inflammatory markers such as elevated levels of blood interleukin-6, C-reactive protein, fibrinogen, and decreased albumin levels,^25^ which impairs glucose stability and β-cell function, and increases the risk of T2DM.^26^ Furthermore, daytime napping can induce non-rapid eye movement sleep^27^ and disturb circadian rhythms, resulting in fluctuation of glucose tolerance.^28^ Long sleep duration and daytime napping can be caused by sleep apnea.^26^^,^^29^ As previously reported, Asians are more likely to experience obstructive sleep apnea than Western populations, regardless of BMI.^30^ Sleep apnea is a respiratory dysfunction that presents with snoring and hypoxia,^31^ both of which increase sympathetic nervous activity and hormone dynamics like cortisol, resulting in insulin resistance^32^ and the incidence of T2DM.^33^

The strengths of the present study are its prospective design, a large number of incident T2DM cases, and comprehensive surveys to examine multiple confounding factors. However, this study had several limitations. First, we used self-reported physician-diagnosed diabetes, which may be prone to misclassification. However, the sensitivity and specificity of self-reporting compared to participants’ glucose concentrations or treatments were relatively high in the validation study, with a sensitivity of 70% for men and 75% for women, and specificity of 95% for men and 98% for women.^23^ Second, we lacked detailed information on sleep and daytime napping, such as sleep quality, sleep apnea, and nap duration. Although we could only examine the impacts of daytime napping, a recent meta-analysis of cohort studies demonstrated that long daytime napping of one hour or more was associated with an increased risk of incident T2DM (risk ratio 1.31; 95% CI, 1.02–1.67), while such an association was not found for short daytime napping of less than one hour (risk ratio 1.06; 95% CI, 0.79–1.42).^13^ Further study focusing on the duration of daytime napping are necessary to conduct a more detailed evaluation of the impact of daytime napping. Third, because data on sleep duration were obtained from self-administrated questionnaires, it may result in misclassification. However, self-reported sleep duration has been shown to correlate well with quantitative sleep assessment with actigraphy.^34^ Therefore, the effect of misclassification in the present study can be limited. Finally, due to the two-point assessment of diabetes, at baseline and 5 years later, the present study design is weaker than a yearly assessment, and we cannot fully estimate the incidence of diabetes during the follow-up.

### Conclusion

Sleep duration of 10 hours or more was associated with a two-times higher risk of T2DM compared to 7 hours of sleep in both men and women, and this association was confined to the non-overweight. The risks of T2DM were 2.5 to 3 times higher in nappers with the long sleep duration than in non-nappers without it.
